# Exploring the Determinants of Nascent Social Entrepreneurial Behaviour

**DOI:** 10.3390/ijerph19063556

**Published:** 2022-03-17

**Authors:** Ching Yin Ip, Tingna Zhuge, Yu Shan Chang, Ting-Huei Huang, Yin-Lin Chen

**Affiliations:** Department of Advertising and Public Relations, Fu Jen Catholic University, New Taipei City 242062, Taiwan; 408070069@mail.fju.edu.tw (T.Z.); 408070411@gapp.fju.edu.tw (Y.S.C.); 407560251@gapp.fju.edu.tw (T.-H.H.); 408012536@mail.fju.edu.tw (Y.-L.C.)

**Keywords:** empathy, experience, outcome expectations, self-efficacy, social entrepreneurial behaviour, social support

## Abstract

Following the emergence of social, environmental, and public health issues, this study analysed the determinants of nascent social entrepreneurial behaviour. This research investigated the effects of empathy and prior experience with social problems on nascent social entrepreneurial behaviour through social entrepreneurial self-efficacy, outcome expectations of social entrepreneurship, and perceived social support. Through utilising the quantitative survey method, this study gathered a total of 560 valid responses, comprising 258 Chinese respondents and 302 Taiwanese respondents. Outcome expectations and perceived social support positively influenced nascent social entrepreneurial behaviour for the Chinese sample, whereas social entrepreneurial self-efficacy and perceived social support positively influenced nascent social entrepreneurial behaviour for the Taiwanese sample. This research enriches the existing literature by taking account of social entrepreneurial behaviour, instead of merely analysing social entrepreneurial intentions.

## 1. Introduction

Governments and societies are increasingly aware of different social and environmental problems such as global warming, rural poverty, and ageing population, as well as public health challenges such as alcohol-related harms, food safety, substance abuse, and teen pregnancy. Notably, the United Nations has proposed Sustainable Development Goals (SDGs), which signify the importance of implementing strategies for tackling different social problems such as eradicating poverty, improving health and education, reducing inequality, and promoting environmentally sustainable practices. Social entrepreneurship, which connects its social mission to entrepreneurial activities [1], can act as an innovative and sustainable means for relieving social problems without relying on donations and posing a burden to the government. Therefore, it is critical to put more effort into educating the public to recognise the values of social entrepreneurship and attracting young people to engage in social entrepreneurship.

Prior studies have focussed on analysing the determinants of social entrepreneurial intentions [2,3,4,5,6,7]. These studies have identified and empirically tested the influence of empathy, perceived social support, moral obligation, prior experience with social problems, outcome expectations of social entrepreneurship, and social entrepreneurial self-efficacy on social entrepreneurial intentions [3,4,5]. It was argued that evaluating the antecedents of entrepreneurial intentions could help to predict entrepreneurial behaviour and the process of business creation [8]. Although the theory of planned behaviour signifies that attitude, subjective norms, and perceived behavioural control can generate intentions and behaviour [9], a majority of studies on social entrepreneurial intentions taking account of this theory have neglected the role of social entrepreneurial behaviour by simply assuming that intentions can lead to behaviour. Although certain research has displayed the positive association between entrepreneurial intentions and nascent entrepreneurial behaviour [10], entrepreneurial intentions may not always lead to entrepreneurial behaviours because of the potential for action aversion and action doubt [11]. In order to bridge this gap, it is critical to explore and identify the determinants of nascent social entrepreneurial behaviour and revisit the applicability of variables tested in social entrepreneurial intentions models.

Social entrepreneurship research in East Asia is emerging. For example, because the Taiwanese government started recognising the importance of promoting social innovation and social entrepreneurship, including the establishment of Social Innovation Lab and the implementation of Social Innovation Action Plan, the public is increasingly aware of social entrepreneurship [12]. More and more studies in Taiwan have evaluated the determinants of social entrepreneurial intentions of different respondents, such as Taiwanese journalists and students [1,7]. Research on social entrepreneurial intentions in the East Asian context displayed that prior experience and perceived social support were the critical predictors of social entrepreneurial intentions, which echoes with the emphasis on Oriental collectivist values in maintaining social harmony and receiving social support [4,7]. In addition, empathy was found to be an antecedent of social entrepreneurial self-efficacy and social entrepreneurial intentions in samples of Hong Kong students, Taiwanese journalists, and Taiwanese residents [4,5,7]. A recent study further discovered the positive effect of outcome expectations of social entrepreneurship on social entrepreneurial intentions based on social cognitive career theory [5].

Consequently, there were three objectives for this study, including (a) to operationalise nascent social entrepreneurial behaviour, (b) to evaluate the effects of empathy, prior experience, social entrepreneurial self-efficacy, outcome expectations, and perceived social support on nascent social entrepreneurial behaviour, (c) to analyse whether the results are consistent in both Chinese and Taiwanese samples, and (d) to offer suggestions in fostering social entrepreneurship.

## 2. Literature Review

### 2.1. Social Entrepreneurship and Nascent Social Entrepreneurial Behaviour

Because diverse of definitions of social entrepreneurship have been proposed, there is no consensus on its definition. Social entrepreneurship was defined based on the contexts and sectors that each researcher was interested in; thus, social entrepreneurship has become a multi-interpretable concept, which can refer to sectors of not-for-profit, for-profit, or hybrid form, or even entrepreneurship regardless of the above forms [13]. Some considered social entrepreneurship as not-for-profit and a part of not-for-profit organisations which engage in entrepreneurially virtuous behaviour to commit to social missions and recognise social entrepreneurial opportunities [14]. Others adopted the for-profit form of social entrepreneurship and regarded social entrepreneurship as a measure for addressing social missions while generating profits through social entrepreneurial activities and social innovation, in order to ensure venture sustainability [15]. Robinson [16] emphasised the hybrid form of social entrepreneurship and defined the term as “the identification of a specific social problem and a specific solution (or set of solutions) to address it; the evaluation of the social impact, the business model and the sustainability of the venture; and the creation of a social mission-oriented for-profit or a business-oriented non-profit entity that pursues the double (or triple) bottom line” (p. 95). In order to distinguish social enterprises from non-profit organisations which rely on donations, Ip and Liang’s [15] definition is utilised in this study.

A social enterprise is considered as an enterprise with a mission to relieve social problems while adopting business models and generating profits through social innovation for venture sustainability and reinvestment [15]. Considering that entrepreneurial behaviour is the know-how or knowledge which allows an individual to perform certain actions [17], this study conceptualises nascent social entrepreneurial behaviour as the know-how, experience, and knowledge which allow an individual to be prepared to establish a social enterprise, such as the ability to conduct market research that can assist the development of products or services of a social enterprise and the ability to develop a social enterprise business plan. More emphasis on marketing and financial management should be placed because this can become an obstacle for social venture survival [18]. Research indicated that entrepreneurial self-efficacy, entrepreneurial intentions, and external factors such as education and family background can generate the likelihood to take entrepreneurial action [17,19,20]; however, most of these studies focussed on commercial enterprises instead of social enterprises. The lack of research on social entrepreneurial behaviour urges exploration of the critical elements that foster social entrepreneurial behaviour and action.

### 2.2. Theoretical Foundation of Analysing Nascent Social Entrepreneurial Behaviour

A majority of studies on social entrepreneurial intentions referred to the variables proposed by Mair and Noboa [21] and Hockerts [3], which were based on the theory of planned behaviour. The theory of planned behaviour signifies that attitude, subjective norms, and perceived behavioural control can generate intentions and behaviour [9]. Mair and Noboa [21] proposed that empathy was a proxy of attitude, moral judgement was a proxy of subjective norm, self-efficacy was a proxy of perceived internal behavioural control, and social support was a proxy of perceived external behavioural control. Hockerts [3] empirically tested the model, which was revisited by several studies [4,6,7], displaying the importance of prior experience with social problems, social entrepreneurial self-efficacy, and perceived social support in enhancing social entrepreneurial intentions. Taking account of social cognitive career theory, Ip et al. [5] further incorporated outcome expectations of social entrepreneurship and revealed a positive influence on social entrepreneurial intentions. Although social entrepreneurial intentions have been tested thoroughly, research regarding the social entrepreneurial intentions of a behaviour process remains limited [22]. Therefore, social entrepreneurial behaviour should also be operationalised and investigated instead of simply assuming the positive association between intentions and behaviour. Referring to the aforementioned studies, empathy, prior experience with social problems, social entrepreneurial self-efficacy, outcome expectations, and perceived social support were adopted and tested as the determinants of nascent social entrepreneurial behaviour.

Moreover, neglecting the consideration of nascent social entrepreneurial behaviour is criticised based on three major reasons. First, research showed that entrepreneurial intentions explained only less than one-third of the variance in entrepreneurial behaviour [10], implying the need to explore alternative determinants of entrepreneurial behaviour. Research also discovered that the effect of entrepreneurial intentions on entrepreneurial behaviour was not strong [23]. Notably, there is not enough evidence that the determinants of social entrepreneurial intentions are also applicable for generating social entrepreneurial behaviour. Second, the intention–action gap suggests that intentions are postponed or abandoned because of the emergence of different constraints such as action aversion and action doubt, or changes in a person’s preferences [11]. Hence, the assumption of entrepreneurial intentions leading to entrepreneurial behaviours may not always be tenable. Third, although many studies of social entrepreneurial intentions referred to the theory of planned behaviour [3,4,6,7], these researchers have not actually considered and tested social entrepreneurial behaviours. For example, Ip et al. [5] displayed the effects of prior experience, empathy, social entrepreneurial self-efficacy, perceived social support, social responsibility, and outcome expectations on social entrepreneurial intentions; however, the empirical model did not incorporate social entrepreneurial behaviour. Therefore, these reasons can serve as the foundation and research gap for exploring the determinants of social entrepreneurial behaviour.

### 2.3. Determinants of Nascent Social Entrepreneurial Behaviour

Social entrepreneurial self-efficacy is regarded as an individual’s belief in their capability to contribute to the relief of social problems [3]. The positive association between social entrepreneurial self-efficacy and social entrepreneurial intentions has been supported in different samples, such as Mexico, Philippines, and Taiwan [5,24,25,26]. Because self-efficacy is associated with greater levels of expandability, innovativeness, social impact, and higher potential for venture survival, social entrepreneurs should develop self-efficacy beliefs in order to adapt to the social entrepreneurship environment [27]. Research on a sample from a developing country has reported a positive influence of self-efficacy on social entrepreneurial behaviour [28]. Moreover, because entrepreneurial self-efficacy implies the ability to start a new project and engage in entrepreneurial activities, results from a Chinese sample have indicated that people possessing a higher level of entrepreneurial self-efficacy are more likely to take entrepreneurial action [29]. Therefore, those who perceive a high potential of failure in starting a business will be less likely to engage in business activities [30]; similarly, those who believe that they do not possess the capability in dealing with social problems will be unlikely to take social entrepreneurial action. The following hypothesis is then proposed:

**Hypothesis** **1** **(H1).** *Social entrepreneurial self-efficacy positively affects nascent social entrepreneurial behaviour.*

Outcome expectations of social entrepreneurship are conceptualised as the perceived consequences and efficacy of starting a social enterprise [5]. Social cognitive career theory signifies the importance of social cognitive factors including self-efficacy and outcome expectations in shaping an individual’s career goals [31]; indeed, the theory has been applied to both conventional and social entrepreneurship contexts, which discovered the positive association between outcome expectations and entrepreneurial intentions [5,32]. Even when self-efficacy has been controlled, outcome expectations are still found to generate entrepreneurial intentions [32], showing that managing awareness of the consequences of engaging in social entrepreneurship is a vital element in fostering social entrepreneurial interests. Because there are different ways to deal with social problems (e.g., donating to charity, working in a non-profit organisation, and conventional businesses engaging in corporate social responsibility), an individual should recognise the advantages of social entrepreneurship in dealing with emerging social problems before taking further action [5]. Despite the lack of research on social entrepreneurial behaviour, the following hypothesis deserves further investigation:

**Hypothesis** **2** **(H2).** *Outcome expectations positively affect nascent social entrepreneurial behaviour.*

Perceived social support is regarded as the personal belief in the actual and potential resources acquired from a social network [33]. The positive effect of perceived social support on social entrepreneurial intentions is generally consistent in previous studies [3,4,5,7]. Social support provides both the emotional and instrumental support which are necessary for business start-ups, including encouragement and approval to become a social entrepreneur and accurate and relevant information which facilitates the establishment and operation of a social enterprise [34]. In addition, it was found that social capital could enhance entrepreneurial passion and develop the capabilities to become an entrepreneur, which fostered engagement in nascent entrepreneurial behaviours [35]. Most importantly, acquiring social support is a vital element before making decisions and taking action in the East Asian context because the collectivistic values can lead to reliance on opinions from peers and family members [36]. Therefore, perceived social support should not be neglected in analysing social entrepreneurial behaviours, and the following hypothesis is proposed:

**Hypothesis** **3** **(H3).** *Perceived social support positively affects nascent social entrepreneurial behaviour.*

Empathy is defined as “the ability to intellectually recognise and emotionally share the emotions or feelings of others” [21] (p. 128). Empathy is suggested as being able to nurture one’s capabilities in recognising consumers’ needs, fostering social innovation, tackling workplace stress, and motivating employees [37,38], which are essential in starting and operating a social enterprise. Because empathy can also enhance the desire and passion to take action in contributing to society [39], empathetic individuals may be more aware of the different means for relieving social problems and may recognise the social and economic value of social entrepreneurship. Consequently, they may form favourable attitude towards social entrepreneurship or even starting a social enterprise. Moreover, empathy can facilitate the emergence of reciprocal relationships because empathetic individuals tend to be more aware of others’ feelings [40]. Indeed, people strive to maintain a balance between the amount of social support they receive and give, in order to prevent feeling guilty if they did not reciprocate; hence, those who offer more help tend to receive more social support in reciprocity [40].

Research on a university student sample displayed that empathy (in dimensions of perspective-taking and empathic concern) indirectly affected social entrepreneurial intentions through social entrepreneurial self-efficacy and that there was no significant direct effect of empathy on social entrepreneurial intentions [41]. Empathetic individuals are more equipped to realise what others in need are facing and what they may need, as well as feeling more confident in becoming a social entrepreneur [41]. A recent empirical study in Taiwan also reported a positive indirect effect of empathy on outcome expectations through social entrepreneurial self-efficacy [5]. Despite the lack of research on nascent social entrepreneurial behaviour, the present research posits the indirect effect of empathy on nascent social entrepreneurial behaviour. The following hypothesis is proposed:

**Hypothesis** **4** **(H4).** *Empathy positively affects (a) social entrepreneurial self-efficacy, (b) outcome expectations, and (c) perceived social support.*

Prior experience with social problems is regarded as the extent of an individual’s practical experience and knowledge in working with social organisations and dealing with social problems [3]. The literature on entrepreneurial intentions displayed the effect of prior experience on entrepreneurial intentions and behaviours. For example, because entrepreneurial experience provides opportunities to learn from role models, people with more entrepreneurial experience are more competent when taking action in starting a business [42]. Notably, young adults can accumulate entrepreneurial experience from education and work, in order to acquire necessary tangible and intangible resources (such as financial capital, knowledge, and social capital) for engaging in start-up behaviour, supporting the association between entrepreneurial experience and entrepreneurial behaviour [43].

In the social entrepreneurship context, contextual factors such as education and experience are proposed as improving outcome expectations of engaging in social entrepreneurship and social entrepreneurial self-efficacy, which can develop social entrepreneurial intentions [44]. Moreover, Hockerts [3] discovered that prior experience with social problems generated both social entrepreneurial self-efficacy and perceived social support, which in turn enhanced social entrepreneurial intentions because the experience can offer knowledge and skills for relieving social problems. Notably, an indirect effect of social entrepreneurial self-efficacy on Islamic social entrepreneurial intentions of university students in Bangladesh was also found [45]. Recent empirical research in Taiwan further reported that prior experience with social problems fostered social entrepreneurial self-efficacy, outcome expectations, and perceived social support, which nurtured people’s interests in starting a social enterprise [5]. Despite the lack of research on nascent social entrepreneurial behaviour, the present study also posits the indirect effect of prior experience with social problems on nascent social entrepreneurial behaviour. The following hypotheses are proposed:

**Hypothesis** **5** **(H5).** *Prior experience with social problems positively affects (a) social entrepreneurial self-efficacy, (b) outcome expectations, and (c) perceived social support.*

**Hypothesis** **6** **(H6).** *Empathy indirectly affects nascent social entrepreneurial behaviour through social entrepreneurial self-efficacy, outcome expectations, and perceived social support.*

**Hypothesis** **7** **(H7).** *Prior experience with social problems indirectly affects nascent social entrepreneurial behaviour through social entrepreneurial self-efficacy, outcome expectations, and perceived social support.*

**Figure 1:**
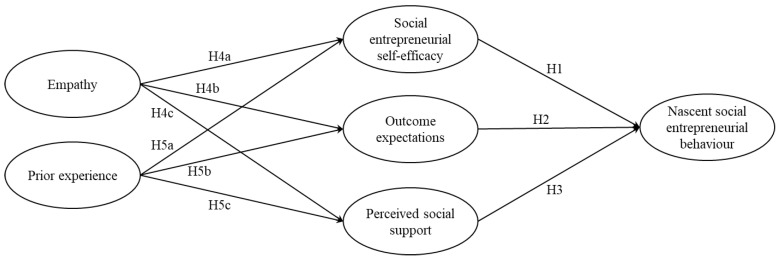
Determinants of nascent social entrepreneurial behaviour. Note: H6 and H7 represent indirect effects.

Therefore, the following conceptual model is proposed (Figure 1).

## 3. Data and Methods

### 3.1. Sample and Procedures

A survey was designed in order to investigate the determinants of nascent social entrepreneurial behaviour of Chinese and Taiwanese residents. The survey was posted on online platforms during November to December 2021, including forums (e.g., ptt and dcard) and social media (e.g., Facebook and Line) in gathering a Taiwanese sample, as well as WeChat and Weibo in gathering a Chinese sample. After collecting the samples, a confirmatory factor analysis (CFA) was performed using Amos 27.0, in order to validate the factor structure of the six constructs. Convergent validity and item consistencies were tested by standardised factor loadings, composite reliability (CR), and average variance extracted (AVE). In addition, discriminant validity was evaluated by using heterotrait–monotrait correlations (HTMT) [46]. Following the CFA analysis, two structural models using the maximum likelihood method were adopted to test the hypotheses of the proposed model for the Chinese sample and Taiwanese sample. Moreover, the indirect effects were tested using 5000 bootstrap samples and 95% confidence intervals (CI).

The study has gathered a total of 560 valid responses, including 258 Chinese respondents and 302 Taiwanese respondents. The sample constituted 185 (33.0%) men and 375 (67.0%) women. A majority of respondents were 21 to 30 years old (46.8%), followed by 20 years old or below (25.5%), 41 to 50 years old (14.8%), 51 years old or above (8.4%), and 31 to 40 years old (4.5%). For highest level of education, a majority of respondents possessed an undergraduate degree (85.2%), followed by having a postgraduate degree (7.5%), and having a high school diploma (7.3%).

### 3.2. Measures

This study referred to scales adopted in previous studies. Three items each of empathy, prior experience, social entrepreneurial self-efficacy, and perceived social support referred to Hockerts [3]. Four items on outcome expectations of social entrepreneurship originated in Ip et al. [5]. Five items were used to measure nascent social entrepreneurial behaviour and were adapted from Gieure et al. [17]. All items were measured on a 6-point Likert scale ranging from 1 (strongly disagree) to 6 (strongly agree). Survey items are shown in the Appendix A.

## 4. Results

### 4.1. Measurement Model

A CFA was first adopted to evaluate the factor structure of variables on the full sample of 560 respondents (Table 1). The hypothesised CFA model yielded an acceptable fit (*χ*^2^/*df* = 3.28, CFI = 0.94, TLI = 0.93, RMSEA = 0.06, SRMR = 0.06) [47]. Because standardised factor loadings were larger than 0.5, CRs were larger than 0.6, and AVEs were larger than 0.5, convergent validity was achieved [48,49]. Moreover, discriminant validity was also achieved because all HTMT pairs were lower than the strict 0.85 threshold [46]. The correlation matrix and HTMT matrix are displayed in Table 2 and Table 3, respectively.

### 4.2. Structural Model

**Figure 2:**
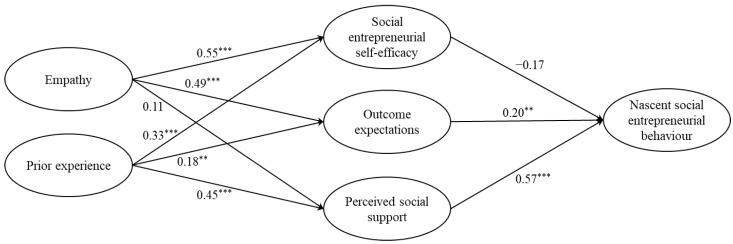
Results of the hypothesised structural model for Chinese respondents. ** *p* < 0.01, *** *p* < 0.001.

**Figure 3:**
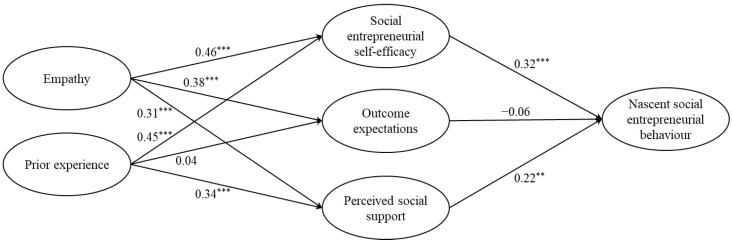
Results of the hypothesised structural model for Taiwanese respondents. ** *p* < 0.01, *** *p* < 0.001.

After showing satisfactory indicators of convergent and discriminant validity from the CFA, the hypothesised structural model was then tested for Chinese respondents and Taiwanese respondents in order to evaluate the consistency of variables contributing to nascent social entrepreneurial behaviour. Results indicate that both the hypothesised structural model for Chinese respondents (*χ*^2^/*df* = 2.08, CFI = 0.94, TLI = 0.93, RMSEA = 0.07, SRMR = 0.07) and Taiwanese respondents (*χ*^2^/*df* = 2.43, CFI = 0.93, TLI = 0.92, RMSEA = 0.07, SRMR = 0.08) also yielded an acceptable fit [47]. The structural models with standardised beta values for Chinese respondents and Taiwanese respondents are displayed in Figure 2 and Figure 3, respectively.

Results of the Chinese sample are first evaluated (Figure 2). Social entrepreneurial self-efficacy had no significant effect on nascent social entrepreneurial behaviour (*β* = −0.17, *SE* = 0.14, *p* > 0.05), rejecting H1. Outcome expectations positively affected nascent social entrepreneurial behaviour (*β* = 0.20, *SE* = 0.07, *p* < 0.01), supporting H2. Perceived social support positively affected nascent social entrepreneurial behaviour (*β* = 0.57, *SE* = 0.11, *p* < 0.001), supporting H3. Empathy positively affected social entrepreneurial self-efficacy (*β* = 0.55, *SE* = 0.10, *p* < 0.001) and outcome expectations (*β* = 0.49, *SE* = 0.10, *p* < 0.001), but had no significant effect on perceived social support (*β* = 0.11, *SE* = 0.12, *p* > 0.05); hence, H4a and H4b were supported, whereas H4c was rejected. Prior experience positively affected social entrepreneurial self-efficacy (*β* = 0.33, *SE* = 0.05, *p* < 0.001), outcome expectations (*β* = 0.18, *SE* = 0.06, *p* < 0.01), and perceived social support (*β* = 0.45, *SE* = 0.08, *p* < 0.001), which supported H5a, H5b, and H5c. For the indirect effects, empathy had no significant indirect effect on nascent social entrepreneurial behaviour through social entrepreneurial self-efficacy, outcome expectations, and perceived social support (*β* = 0.07, *SE* = 0.08, *p* > 0.05, 95% CI (−0.09, 0.21)), rejecting H6. However, prior experience positively affected nascent social entrepreneurial behaviour through social entrepreneurial self-efficacy, outcome expectations, and perceived social support (*β* = 0.24, *SE* = 0.06, *p* < 0.001, 95% CI (0.13, 0.35)), supporting H7.

Results of the Taiwanese sample were also evaluated (Figure 3). Social entrepreneurial self-efficacy positively affected nascent social entrepreneurial behaviour (*β* = 0.32, *SE* = 0.10, *p* < 0.001), supporting H1. Outcome expectations had no significant effect on nascent social entrepreneurial behaviour (*β* = −0.06, *SE* = 0.08, *p* > 0.05), rejecting H2. Perceived social support positively affected nascent social entrepreneurial behaviour (*β* = 0.22, *SE* = 0.09, *p* < 0.01), supporting H3. Empathy positively affected social entrepreneurial self-efficacy (*β* = 0.46, *SE* = 0.09, *p* < 0.001), outcome expectations (*β* = 0.38, *SE* = 0.09, *p* < 0.001), and perceived social support (*β* = 0.31, *SE* = 0.10, *p* < 0.001), which support H4a, H4b, and H4c. Prior experience positively affected social entrepreneurial self-efficacy (*β* = 0.45, *SE* = 0.07, *p* < 0.001), and perceived social support (*β* = 0.34, *SE* = 0.07, *p* < 0.001) but had no significant effect on outcome expectations (*β* = 0.04, *SE* = 0.06, *p* > 0.05); hence, H5a and H5c were supported whereas H5b was rejected. For the indirect effects, empathy positively affected nascent social entrepreneurial behaviour through social entrepreneurial self-efficacy, outcome expectations, and perceived social support (*β* = 0.19, *SE* = 0.04, *p* < 0.001, 95% CI (0.11, 0.28)), supporting H6. Moreover, prior experience positively affected nascent social entrepreneurial behaviour through social entrepreneurial self-efficacy, outcome expectations, and perceived social support (*β* = 0.21, *SE* = 0.06, *p* < 0.001, 95% CI (0.10, 0.33)), supporting H7. The summary of results is displayed in Table 4.

## 5. Discussion

This study offers several theoretical and practical implications. First, an operationalisation of nascent social entrepreneurial behaviour was developed and empirically tested. This contributes to the social entrepreneurship literature which ignored the role of social entrepreneurial behaviour as suggested in the theory of planned behaviour [3,5]. This also extends existing studies which assumed that social entrepreneurial intentions would lead to behaviour, thereby not empirically testing behaviour [3,5,7]. Second, apart from prior studies reporting the weak association between entrepreneurial intentions and entrepreneurial behaviour [23], the results in this study also indicate certain differences when compared with studies on social entrepreneurial intentions. For example, the effect of social entrepreneurial self-efficacy was stronger than the effect of perceived social support on nascent social entrepreneurial behaviour for the Taiwanese sample, which is different from results in social entrepreneurial intentions studies displaying perceived social support as the most prominent factor [4,7]. In addition, differences in the effects of outcome expectations and social entrepreneurial self-efficacy on nascent social entrepreneurial behaviour in both samples may imply the existence of cultural influences; hence, this offers the ground for future studies to evaluate the potential impact of cultural factors. Third, through exploring the effects of empathy, prior experience, social entrepreneurial self-efficacy, outcome expectations, and perceived social support on nascent social entrepreneurial behaviour, it was found that prior experience and perceived social support are still the consistent predictors of behaviour. This provides a foundation for future studies in taking account of these variables when analysing the determinants of nascent social entrepreneurial behaviour, especially within the East Asian context. Fourth, practical implications can be provided to educators and government officials. In Taiwan, different courses or programs of social entrepreneurship are offered to students, including Fu Jen Catholic University, which has established a master’s program in social enterprise with a view to nurturing students’ capabilities and interests in starting a social enterprise. Therefore, the results of this study can offer insights for teachers to design activities in equipping students’ experience and capabilities when dealing with social problems and providing more opportunities for students to develop a diverse social network through group work and mentorship programs. Considering that self-efficacy is not a fixed trait [31], government officials can also initiate legislation or policies which can remove the barriers for potential social entrepreneurs when starting a social enterprise [5].

Results from the two samples indicate that perceived social support is a significant predictor of nascent social entrepreneurial behaviour, which echoes prior studies showing the critical role of perceived social support on social entrepreneurial intentions in Northeast Asian samples [4,5,7]. Interestingly, the effect was even stronger for the Chinese sample. A possible reason could be the collective tendency and political reality in mainland China [1], because the Chinese emphasise social harmony and group values, in addition to compliance with the norms of the Chinese government and society in operating a venture. Hence, Chinese respondents tend to be more pragmatic before making a decision to start a business and tend to rely more on others’ opinions and assistance in order to reduce the barriers in starting and operating a social enterprise. Therefore, developing social networks remains an important factor for encouraging people to engage in social entrepreneurship. Educators should organise more internship and mentorship programs in order to offer opportunities for students to establish a diverse social network which can facilitate the emergence of innovative ideas and financial support. A good example is the establishment of the social innovation lab by the Taiwanese government, which provides mentorship programs and social networking opportunities for social entrepreneurs.

The effect of outcome expectations on nascent social entrepreneurial behaviour was rather different between the two samples. Only the Chinese sample reported a significant positive effect, whereas the Taiwanese sample reported a nonsignificant effect, which is different from results from recent empirical research in Taiwan [5]. A possible reason could be that Ip and colleagues’ [5] research only considered social entrepreneurial intentions, without incorporating social entrepreneurial behaviour. This indicates that although some Taiwanese respondents recognise the advantages of social entrepreneurship in relieving social problems and are interested in becoming social entrepreneurs, they may not be ready to take action in establishing a social enterprise. For instance, young adults may not yet have enough knowledge, experience, and social support in running a business and writing a business proposal. Instead, results from the Chinese sample echo prior studies indicating a significant positive effect of outcome expectations on social entrepreneurial intentions [5,32]. A possible reason could be that Chinese residents enjoy relatively low operating costs and the enormous spurt of China’s e-commerce sector, which greatly reduces the obstacles to taking social entrepreneurial action. On the whole, the results of this study received partial support from social cognitive career theory, which suggested that both self-efficacy and outcome expectations served as critical elements contributing to career interests and goals [31]. In contrast with the theory’s propositions, a recent longitudinal study also discovered that outcome expectations and entrepreneurial self-efficacy did not directly influence nascent entrepreneurial behaviour [50]. This may imply that the transformation of favourable outcome expectations into behaviour is complex, and more efforts are required to initiate a favourable environment for the emergence of social entrepreneurship. Instead of just educating on social entrepreneurship or social problems [44], educators should also initiate classroom discussions that identify and compare different alternatives for relieving social problems, including conventional business engaging in corporate social responsibility and non-profit organisations in helping the disadvantaged. Afterwards, students could decide whether social entrepreneurship is a better option for tackling social problems. Moreover, government officials may collaborate with opinion leaders in promoting social entrepreneurship in order to generate public attention towards social entrepreneurship.

The effect of social entrepreneurial self-efficacy on nascent social entrepreneurial behaviour was only supported in the Taiwanese sample. This echoes another study in the Taiwanese context, reporting a positive effect on social entrepreneurial intentions [5]. However, the findings are different from a prior study on a Chinese sample which reported that people having a higher level of entrepreneurial self-efficacy are more likely to take entrepreneurial action [29]. A possible reason could be that the Taiwanese sample was dominated by young adults, whereas the Chinese sample was relatively mature. Because those who are more mature are implied to have more working or entrepreneurship experience, they may be more pragmatic in deciding to engage in social entrepreneurial behaviour (i.e., put more emphasis on gaining economic revenue for venture survival), instead of prioritising dealing with social problems and creating social values (i.e., the essence of social entrepreneurial self-efficacy). Even though social entrepreneurship addresses creation of social values, attaining economic goals is still vital to venture survival because selling products or services innovatively and viably is a prerequisite for financial sustainability of a social enterprise [51]. In order to enhance social entrepreneurial self-efficacy, educators should also encourage students to transform an innovative idea into action—for example, through participating in social business plan competitions. In addition, governments can take the initiative to reduce the barriers for potential social entrepreneurs in starting or operating a social venture [5] by implementing education policy to raise public attention and awareness towards social and environmental problems, implementing a tax reduction for social enterprises, and establishing a legal framework for social enterprises.

Although results from the two samples display a significant influence of empathy on social entrepreneurial self-efficacy, which is consistent with a previous study [41], the indirect effect of empathy was only supported in the Taiwanese sample. A major reason could also be explained by the pragmatic view of Chinese respondents, because empathetic individuals may still choose to volunteer or give donations [26], instead of deciding and taking action to become social entrepreneurs. Due to the insider–outsider distinction, a study also indicated the inclination of people in practicing empathy only with those that they are familiar with rather than strangers [52]. Because social entrepreneurship requires continuing effort, Chinese residents may adopt more convenient and effortless measures for contributing to society. Consequently, they may engage in social entrepreneurial behaviour only if they possess enough related experience and social resources which are necessary for venture start-up and survival. However, taking account of the positive indirect effect of empathy in the Taiwanese sample, measures to foster empathy should also be proposed. Educators can utilise simulation activities (e.g., reflecting the difficulties encountered by the disadvantaged and the consequences of environmental problems) to encourage students to recognise different social problems and understand others’ feelings. Because effective listening serves as an important element in nurturing an individual’s sense of empathy, incorporating service learning into curricula may also help to equip students’ listening and interpretation capabilities [53].

The indirect effect of prior experience on nascent social entrepreneurial behaviour was supported in both samples, which is consistent with prior studies on social entrepreneurial intentions [3,5]. Because prior experience offers the skills in operating a business, develops the ability to identify market opportunities, establishes social networks, and generates interests in starting a social enterprise [54,55], this can nurture an individual to be competent to engage in social entrepreneurial behaviour. Therefore, schools should encourage students to participate in volunteer work [5] through offering extra credits or scholarships to participants. Governments may also implement policies for motivating schools and conventional businesses to establish volunteering programs in order to strengthen public awareness towards social problems.

The study has several limitations. First, due to the obstacles in collecting enough data in mainland China, the survey was mainly distributed through WeChat by adopting snowball sampling. Therefore, generalising results in this study to the Chinese population should be dealt with care. Further analyses in revisiting this model may be necessary to offer more convincing evidence. Second, in order to ensure the conciseness of the survey, items regarding social entrepreneurial intentions were not incorporated. Future research should also consider social entrepreneurial intentions in order to investigate the path between social entrepreneurial intentions and behaviour. Third, due to the contradictory results of the effect of subjective norms on social entrepreneurial intentions [3,4,6,7,45], this study did not incorporate variables signifying subjective norms. Future research should further identify a new proxy for subjective norms [1] and should empirically test their effect on social entrepreneurial intentions and behaviour.

## 6. Conclusions

This study makes three major contributions. First, this study is among the pioneers in operationalising and empirically testing nascent social entrepreneurial behaviour. This offers a foundation on which future studies can investigate the determinants of nascent social entrepreneurial behaviour in different groups, societies, and countries, instead of merely focusing on social entrepreneurial intentions. Second, this research bridges the intention–action gap which indicated the potential for intentions to be postponed or abandoned and to not necessarily transform into behaviour. Because the assumption of intentions leading to behaviours may not always be tenable, researchers should not neglect the role of behaviour as posited in the theory of planned behaviour. Third, this study revisited some variables tested in prior studies of social entrepreneurial intentions [3,5,7] and discovered that these variables may not be applicable in predicting nascent social entrepreneurial behaviour. For example, the effect of social entrepreneurial self-efficacy on behaviour was nonsignificant for the Chinese sample, whereas the effect of outcome expectations on behaviour was nonsignificant for the Taiwanese sample.

Due to the emergence of different social problems followed by the COVID-19 pandemic, as well as the increasing demand for environmental protection, governments and the public should look for viable and alternative means for relieving these problems which may be ignored in societies [56]. Notably, these problems can hardly be tackled by governments and charities alone because the amount of taxation received by governments and donations received by charities decreased during times of economic downturn. Moreover, governments were required to allocate resources to deal with problems with urgent needs (e.g., vaccine development, medical care, and unemployment) during the pandemic, and certain social and environmental problems may usually be ignored under such a critical situation. On the whole, more efforts are needed to encourage people to take action that contributes to societies, such as starting a social enterprise. Emerging applications and research areas such as behaviours of corporate social entrepreneurship [57], or analyses from an institutional level [58], may also deserve further investigation.

## Figures and Tables

**Table 1:** CFA of latent variables (*n* = 560).

Variables and Items	Factor Loadings	*M*	*SD*	CR	AVE
Prior experience with social problems				0.78	0.54
Exp1	0.68	4.01	1.73		
Exp2	0.86	3.67	1.54		
Exp3	0.66	3.69	1.46		
Empathy				0.82	0.61
Emp1	0.68	4.62	1.18		
Emp2	0.84	4.97	1.05		
Emp3	0.80	4.89	1.11		
Social entrepreneurial self-efficacy				0.75	0.51
Eff1	0.79	4.47	1.12		
Eff2	0.72	3.93	1.24		
Eff3	0.63	4.91	1.20		
Perceived social support				0.82	0.62
Sup1	0.87	4.09	1.31		
Sup2	0.89	4.08	1.29		
Sup3	0.55	4.07	1.29		
Outcome expectations of social entrepreneurship				0.90	0.67
Out1	0.80	4.59	1.13		
Out2	0.88	4.56	1.17		
Out3	0.84	4.51	1.15		
Out4	0.79	4.59	1.19		
Nascent social entrepreneurial behaviour				0.92	0.71
Nas1	0.71	2.76	1.50		
Nas2	0.82	2.44	1.50		
Nas3	0.93	2.34	1.49		
Nas4	0.94	2.47	1.52		
Nas5	0.79	2.41	1.51		

**Table 2:** Correlation matrix (*n* = 560).

	1.	2.	3.	4.	5.
1. Experience					
2. Empathy	0.39 ***				
3. Efficacy	0.56 ***	0.69 ***			
4. Support	0.45 ***	0.36 ***	0.74 ***		
5. Outcome	0.29 ***	0.44 ***	0.45 ***	0.46 ***	
6. Behaviour	0.44 ***	0.19 ***	0.44 ***	0.49 ***	0.29 ***

*** *p* < 0.001.

**Table 3:** HTMT matrix (*n* = 560).

	1.	2.	3.	4.	5.
1. Experience					
2. Empathy	0.42				
3. Efficacy	0.56	0.73			
4. Support	0.51	0.43	0.78		
5. Outcome	0.31	0.45	0.47	0.54	
6. Behaviour	0.49	0.23	0.44	0.53	0.29

**Table 4:** Summary of hypotheses.

	Chinese Sample (*n* = 258)		Taiwanese Sample (*n* = 302)	
	Standardised Beta	Result	Standardised Beta	Result
H1: Efficacy → Behaviour	−0.17	Rejected	0.32 ***	Supported
H2: Outcome → Behaviour	0.20 **	Supported	−0.06	Rejected
H3: Support → Behaviour	0.57 ***	Supported	0.22 **	Supported
H4a: Empathy → Efficacy	0.55 ***	Supported	0.46 ***	Supported
H4b: Empathy → Outcome	0.49 ***	Supported	0.38 ***	Supported
H4c: Empathy → Support	0.11	Rejected	0.31 ***	Supported
H5a: Experience → Efficacy	0.33 ***	Supported	0.45 ***	Supported
H5b: Experience → Outcome	0.18 **	Supported	0.04	Rejected
H5c: Experience → Support	0.45 ***	Supported	0.34 ***	Supported
H6: Empathy → Efficacy/Outcome/Support → Behaviour	0.07	Rejected	0.19 ***	Supported
H7: Experience → Efficacy/Outcome/Support → Behaviour	0.24 ***	Supported	0.21 ***	Supported

** *p* < 0.01, *** *p* < 0.001.

## Data Availability

The dataset analysed during the current study is available from the corresponding author on reasonable request.

## Appendix A

**Table A1:** Survey items.

Variables	Items
Prior experience with social problems [3]	Exp1. I have volunteered or otherwise worked with social organisations. Exp2. I have some experience working with social problems. Exp3. I know a lot about social organisations.
Empathy [3]	Emp1. When thinking about socially disadvantaged people, I try to put myself in their shoes. Emp2. Seeing socially disadvantaged people triggers an emotional response in me. Emp3. I feel compassion for socially marginalized people.
Social entrepreneurial self-efficacy [3]	Eff1. I am convinced that I personally can make a contribution to address societal challenges if I put my mind to it. Eff2. I could figure out a way to help solve the problems that society faces. Eff3. Solving societal problems is something each of us can contribute to.
Perceived social support [3]	Sup1. People would socially support me if I wanted to start an organisation to help socially marginalized people. Sup2. If I planned to address a significant societal problem, people would back me up. Sup3. It is possible to attract investors for an organisation that wants to solve social problems.
Outcome expectations [5]	Compared to other means of dealing with social problems, Out1. social entrepreneurship can help the less privileged more effectively. Out2. social entrepreneurship can relieve a social problem more sustainably. Out3. social entrepreneurship can be more able to emphasise a social mission continuously. Out4. social entrepreneurship can draw more public attention to a social problem.
Nascent social entrepreneurial behaviour [17]	Nas1. I have experience in starting new projects or businesses contributing to society. Nas2. I have established a social network that can promote my social venture. Nas3. I prepare to develop a social enterprise business plan. Nas4. I prepare to conduct market research that can assist the development of products or services of a social enterprise. Nas5. I prepare to save money to invest in or start a social venture.
